# How providing a low-cost water filter pitcher led Latino parents to reduce sugar-sweetened beverages and increase their water intake: explanatory qualitative results from the Water Up!@Home intervention trial

**DOI:** 10.1017/S1368980022001744

**Published:** 2022-11

**Authors:** Claudia Santillán-Vázquez, Lucero Hernández, Amanda C Reese, Rosalina Burgos-Gil, Sean D Cleary, Ivonne M Rivera, Joel Gittelsohn, Mark C Edberg, Rafael Monge-Rojas, Uriyoán Colón-Ramos

**Affiliations:** 1 Elliott School of International Affairs, George Washington University, Washington, DC, USA; 2 Milken Institute School of Public Health, George Washington University, 950 New Hampshire Avenue, Washington, DC 20052, USA; 3 CentroNia, Washington, DC, USA; 4 Rivera Group, Hyattsville, MD, USA; 5 Bloomberg School of Public Health, Johns Hopkins University, Baltimore, MD, USA; 6 Costa Rican Institute for Research and Education on Nutrition and Health (INCIENSA), San Jose, Costa Rica

**Keywords:** Sugar-sweetened beverages, Explanatory qualitative interviews, Infants and toddlers, Latino/Hispanic, Tap water

## Abstract

**Objective:**

This study sought to explain results of the Water Up!@Home randomised controlled trial where low-income parents were randomised to receive an educational intervention +a low-cost water filter pitcher or only the filter. Parents in both groups had reported statistically significant reductions in sugar-sweetened beverages (SSB) and increases in water intake post-intervention.

**Design:**

Qualitative explanatory in-depth interviews analysed thematically and deductively.

**Setting:**

Washington, DC metropolitan area, USA.

**Participants:**

Low-income Latino parents of infants/toddlers who had participated in the Water Up! @Home randomised controlled trial.

**Results:**

The filter-stimulated water consumption in both groups by (1) increasing parents’ perception of water safety; (2) acting as a cue to action to drink water; (3) improving the flavour of water (which was linked to perceptions of safety) and (4) increasing the perception that this option was more economical than purchasing bottled water. Safe and palatable drinking water was more accessible and freely available in their homes; participants felt they did not need to ration their water consumption as before. Only intervention participants were able to describe a reduction in SSB intake and described strategies, skills and knowledge gained to reduce SSB intake. Among the comparison group, there was no thematic consensus about changes in SSB or any strategies or skills to reduce SSB intake.

**Conclusions:**

A low-cost water filter facilitated water consumption, which actively (or passively for comparison group) displaced SSB consumption. The findings have implications for understanding and addressing the role of water security on SSB consumption.

Consumption of sugar-sweetened beverages (SSB) is a risk factor for Type 2 diabetes, obesity and other cardiometabolic disorders^(1)^. Consumption of SSB in the USA begins at an early age, with ethnic minorities and low-income groups exhibiting higher prevalence of this behaviour^(2-6)^. As infants and toddlers grow, the cumulative effect of SSB consumption may have detrimental effects on their dietary quality and obesity risk later in life^(7-9)^.

In an advisory report to the Secretary of Health and Human Services and the Secretary of Agriculture, the Dietary Guidelines Advisory Committee recommends individuals to replace SSB consumption with plain water^(10)^. Observational data from the National Health and Nutrition Examination Survey suggest that there is an inverse association between water and SSB consumption among children: those who do not consume water at all consume more energies from SSB^(11)^. Interventions that have aimed to reduce SSB consumption and increase water consumption among children have been found to be, overall, moderately successful if they incorporate nutrition education and work in the home environment^(12)^. Nonetheless, just like in SSB consumption, disparities in water consumption are persistent: overall water consumption remains lowest among younger children, as well as among ethnic and socio-economic minorities^(13,14)^. There is a large body of literature documenting low tap water consumption among minority groups due to mistrust in tap water^(15-20)^, and a study reported that minority groups spend a greater percentage of their household income (up to 16 %) on bottled water compared with non-Hispanic Whites, preventing them from using those funds to buy other things that they need^(21)^. Avoiding tap water has been used as an indicator of water insecurity in the USA and associated with a significantly more SSB consumption on a given day^(22)^. Therefore, recommendations to replace SSB with water face multiple perceived barriers in Hispanic communities where tap water in the USA is perceived to be dirty and unpalatable, and SSB that are perceived to be ‘natural’ are preferable^(23-26)^. A national research agenda to reduce the consumption of SSB and increase safe water access and consumption among 0–5 years old in the USA called for attention to interventions tailored to such high-risk groups, especially if they target fruit-flavoured drinks^(27)^.

In line with this national agenda, our academic-community team developed a theory-based intervention (Water Up!@Home) to replace SSB with water (mostly from the tap, filtered) and reduce excess 100 % juice consumption in a low-income Latino community in the greater DC area^(28)^. Juice consumption was addressed because Hispanic and African-American infants are five to six times as likely as non-Hispanic and Asian infants to exceed paediatric recommendations on 100 % juice consumption^(29,30)^, and our own findings also documented this excess consumption^(28)^. The effects of the intervention were evaluated via a randomised controlled trial that provided families with the intervention (theory-based new curriculum about benefits of water *v*. SSB, plus provision of water filter pitcher) or a water filter pitcher only (without further instructions). Results from the trial suggested that the curriculum plus filter intervention was effective at reducing 100 % fruit juice consumption, while parents and children in both experimental groups significantly increased their overall water consumption and reduced their SSB consumption from baseline^(31)^. These findings raised many questions about the potential effects of the intervention in the context of this community and whether the provision of a water filter alone was enough to replace SSB intake with water consumption in this population.

Therefore, the objective of this manuscript is to understand, from the perspectives of the parents who were exposed to the intervention and those who were in the comparison group, what aspects of the curriculum plus water filter *v*. only providing a water filter may have led them to increase their water intake and reduce SSB for themselves and for their infants/toddlers.

## Methods

This study followed an explanatory qualitative research approach, which is part of a larger mixed-methods study^(32)^. Thirty-two individual, semi-structured, in-depth interviews were conducted in Spanish with Latino parents who were purposively sampled sequentially until saturation was reached among those who received the Water Up!@Home curriculum plus water filter pitcher (twenty-one interviews) and those in the comparison group who only received a water filter pitcher (eleven interviews).

### Brief intervention description

The Water Up!@Home intervention has been described elsewhere^(28)^. In brief, the intervention combined elements of the Health Belief Model and Social Cognitive Theory^(33-35)^, including that health behaviours, are rooted in persons’ perceived susceptibility to a health problem, persons’ perceived severity of the problem, benefits and barriers/costs to behaviour change, ‘cues to action’ and self-efficacy with respect to the behaviour. To address previously documented concerns about the quality and safety of tap water in this community^(24)^, all families in both experimental groups received a National Sanitation Foundation-certified eleven-cup water filter pitcher (∼99 % removal of lead and impurities-not fluoride, with an approximate 2-month lifespan) and an additional filter cartridge. Only families in the intervention group were instructed on proper use and care of water filters and were also provided with a BPA-free infusion water bottle and a child-size pitcher to be used for serving water. The intervention was delivered to families enrolled in the Early Head Start home-visiting programme through three centres in the greater Washington, DC metropolitan area.

### Interview guides

To understand the reasons behind reported behaviour change in beverage consumption, two semi-structured interview guides were designed, one for each group. The guide that was designed to interview intervention parents aimed to elicit rich descriptions about what they liked or disliked about the programme; preferences between consuming bottled *v*. filtered tap water; their thoughts about water and SSB consumption for them and their children; and any differences in consumption behaviours that they noticed before and after receiving the programme. The guide also prompted participants (i.e. ‘*Tell me about the most important things that you learned from the program’)*, and if participants discussed behaviour change, they were probed about self-efficacy in maintaining behaviour change (i.e. ‘*How sure do you feel of maintaining those changes?’).*

The second interview guide reflected the explanatory objective to understand why, when and how participants (who only received the water filter pitcher) may have also changed their beverage consumption. The guide asked participants to describe their views on the health consequences of drinking water and SSB; how and when they acquired that information; their use and their families’ use of the water filter; whether they perceived any changes in what they and their infants/toddlers and other family members ate or drank since receiving the water filter and whether they thought they would be able to sustain consumption of filtered water or any other changes in behaviour that they may have noticed.

Both interview guides were developed and pilot tested in Spanish in order to improve flow and ensure comprehension. They were subsequently refined in terms of wording and eventually translated to English for the purposes of IRB.

### Participant recruitment

Eligible parents were those who self-identified as Hispanic and completed all the follow-up surveys of the randomised controlled trial. A native Spanish-speaking research assistant with extensive experience as a community outreach liaison and trained in public health ethics recruited eligible participants via phone, obtained consent and interviewed them in Spanish via phone or a virtual platform of their choice (due to COVID-19 social distancing recommendations). Based on data we analysed previously and using published guidance^(36)^, we aimed to obtain between 12 and 18 interviews in each group. Our estimate of sample size was eventually confirmed during the analysis – saturation was reached at the twelfth interview for intervention and at the fifth interview for the comparison group.

### Data collection

A bilingual, Latina research assistant with more than 15 years of experience working with parents and other residents in these communities were hired to conduct the in-depth interviews. The senior author and principal investigator trained this research assistant in how to conduct the interviews using an open-ended conversational approach that would allow the participants to feel comfortable sharing their opinions and thoughts, in following systematic techniques for in-depth interviews to allow for flexibility in the discussion, in limiting interviewer persuasion or bias and in encouraging participants to add details and descriptions. Interviews were conducted 1–3 months after the intervention ended; they lasted between 35 and 60 min and were audio-recorded. Participants received a $20 gift card after the interview.

### Data analysis

We used thematic analysis to analyse the data. Braun and Clarke (2006) describe thematic analysis as a method for identifying, analysing and reporting themes within data^(53)^. A theme is a cluster of linked categories conveying similar meanings that reflect meaning within the data^(54)^.

The audio files of all interviews were transcribed verbatim by a professional bilingual transcription service. Transcripts were imported into Atlas.ti and analysed independently by two bilingual (Spanish/English) research assistants who read and coded all transcripts using a codebook previously developed that was based on the intervention’s theoretical framework^(28)^. The same codebook was applied to interviews from the comparison group. Research assistants were instructed to actively identify any newly observed codes and themes that emerged in either group within each domain. After conducting independent analyses, the assistants met to identify any coding discrepancies, noted additional questions or clarifications needed and actively looked for negative cases (descriptions that disagree or differ from the main body of evidence). Further, one of the assistants reviewed all coded paragraphs again to identify which interview questions or discussions prompted each emerging theme and how the participants related the themes to each other. Subsequently, the PI and coinvestigators (including the community partner) met with the research team to resolve any discrepancies in coding, to review any edits to the wording of domains and sub-codes, to group more detailed topics within each domain so as to construct a taxonomy of subcategories and finally to construct an overall picture by exploring the inter-relationships between the various domains. Preliminary thematic findings were then presented to the partnering EHS program staff and home visitors to deepen and expand the understanding of the analyses as a form of member checking. Themes that reached consensus were defined as those that were discussed by more than 80 % of participants. Quotations related to each code were isolated and sorted, generating lists to determine the most common responses. The quotes presented in the results were selected by the team as those that best described each theme and subtheme.

## Results

Results are presented by major topics, themes and subthemes for participants in each group.

### Role of the water filter

There was consensus among participants in both experimental groups that the water filter pitchers motivated them and their children to drink water.

[The filter] motivates you; it motivates you to do it, to accomplish it. (Water Up!@Home participant)

[Speaking of family drinking more water] because you can see how they get excited when they see the cold water in the fridge. (Participant in comparison group)

This motivation to drink water was related to the four subthemes described below.

#### Increased sense of safety of tap water

Knowing where the water came from and seeing the source of the water seemed to be an important consideration for drinking water among participants in both groups. A parent in the comparison group described perceptions of drinking water in their country of origin *v*. the USA to exemplify these perceptions:

There [in the home country] it [water] is natural; one sees that it comes from the earth. Here [in the US] we do not drink pure water. The word ‘pure’ means it comes from a natural spring, where it is sourced. (Participant in comparison group)

Therefore, seeing the filtering process via the water filter pitcher seemed to have increased, at least in part, parents’ perception about the source of the water and that the tap water was safe to drink.

I feel much more secure [with the filter pitcher]. I never drank water from the tap until now that I got the filter (Water Up!@Home participant)

Is the water from the bottles purified? At least now I can see the water going through the filter. (Participant in comparison group)

#### Water filter improved taste of tap water

Participants in both groups explained that the taste of tap water improved with the filter and some associated its taste with their perception of water safety.

One does not know about the pipes where one lives, how old they are, so for me the filter was really important, and in fact, even the water has a better taste. (Water Up!@Home participant)

The water, when you drink it without using the filter, tastes like chlorine but later when you use the filter you can taste the difference (Participant in comparison group)

I feel like it [filter] calls your attention to drink from it because it’s more trustworthy (Participant in comparison group)

#### Using the filter to drink water was more economical than buying bottled water

Participants in both groups also expressed that the filter was more economical than going to the store to buy bottled water. Participants expressed that before participating in this study, they consumed water only if it was purchased in the form of bottled water (often individual, 6 oz. plastic bottles), and that, therefore, they tended to ration their water consumption. With the water filter, they felt that they did not need to ration their water consumption.

I saved money; I saved room; I saved having to work more; surer that I can drink water without having to ration it. (Water Up!@Home participant)

I am happy. I said I am no longer going to spend [money] buying water… I am saving a lot of money. I am no longer spending a lot. Every 15 days I would have to spend $30 [in bottled water]. (Participant in comparison group)

#### The water filter acted as a cue to action to drink more water

Participants in both groups expressed that seeing the water filter cued them and/or their children to drink water.

…to have pure water in front of you, right there at the table… I know that I had to drink the water; [the filter] I had it in front of me and it would tell me ‘it is time for water’ (Water Up!@Home participant)

He [child] is not the type to drink water but since he would see the pitcher there, he would grab it and drink water (Participant in comparison group)

#### Negative cases: persistent mistrust of tap water and convenience of bottled water on the go

Although the filter assuaged some concerns about the safety of tap water, there was still persistent distrust of the tap water among a few participants in both groups

…even though they showed me how to use it, I still wonder if I am using it correctly. (Water Up!@Home participant)

I do not feel that safe… does it really filter the water well/I do not know. I start to think, I don’t feel that safe then. (Participant in comparison group)

These persistent feelings of distrust, in addition to the convenience of bottled water, led participants in both groups to continue purchasing and consuming bottled water, albeit participants in the intervention group explained how they consumed less water from bottled water after the intervention.

We buy fewer bottled water now, my husband takes bottled water with him to work. At least we are drinking filtered water here at home. (Water Up!@Home participant)

### Explaining changes in sugar-sweetened beverages and excess juice consumption observed in quantitative results

Both groups of participants were asked if they had noticed any changes in their beverage consumption (for themselves and for their children) after participating in the study. Parents in the intervention group were able to explicitly describe changes in their consumption patterns (and their children’s) and the strategies that they actively used to achieve that. In contrast, there was no consensus among participants in the comparison group about whether there were any changes in their consumption behaviour since receiving the filter:

Well, I can’t tell you exactly that there was a change… but there could be one for my little girl because it is easier for her to go and grab and drink her cup of water… so maybe it’s a way for her not to drink that much juice. (Participant in comparison group)

It has changed for good…. It is something new for me, for us, the accessibility and the convenience of obtaining clean water (Participant in comparison group)

Before being part of Water Up! I didn’t give her [daughter] water. If I gave her water, it was only like 2 oz and now she drinks more than 2 oz. (Participant in intervention group)

Several subthemes related to strategies introduced in the curriculum intervention emerged only among intervention parents. These included reducing the amount of and were strategies emphasised in the intervention including: added less sugar added to beverages prepared at home; discontinuing the purchases of prepackaged SSB and juice; diluting 100 % juice with plain water; increasing the amount of water consumed; infusing plain water with fresh fruits, herbs or vegetables and incrementally replacing their typical SSB with drinking plain water. These strategies were used to replace their own beverages as well as for their children.

I used to add three spoonfuls of sugar to my coffee and now I only add one, because coffee is [otherwise] too bitter. (Water Up!@Home participant)

If I give him juice, I put more water so that the intensity of the sugar goes down. (Water Up!@Home participant)

When I am having a craving of drinking a soda, I say ‘there is water,’ so then I go and grab my bottle of water and I drink it. (Water Up!@Home participant)

#### Other strategies used by intervention parents to reduce sugar-sweetened beverages consumption

Although the intervention did not suggest homemade beverages as a strategy to reduce SSB or excess juice consumption, this theme emerged when parents in the intervention group explained that as a result of the intervention, they stopped buying prepackaged beverages and focused on preparing these beverages at home, emphasising the ‘natural’ quality of homemade beverages as a benefit over prepackaged drinks.

Especially if it is synthetic, I never buy those juices those Capri Suns and the other little bottles. If I give them something sweet it will be something that I made. (Water Up!@Home participant)

Nonetheless, in these explanations there are some descriptions that suggest that homemade beverages may also include drinks prepared at home with ready-to-mix sugary powders such as Kool Aid or Iced Tea Lemonade.

I stopped buying all of that, prepackaged drinks. I stopped buying all of it. I replaced it [with] things in powdered form… the lemon one or the iced tea. (Water Up!@Home participant)

This is important because although there was no consensus about whether behaviours had changed after participating in the study among comparison parents, this subtheme of ‘natural beverages’ also emerged in this group when they described how they made the decision on what to offer their children.

Orange juice it says they are 100 % natural but who knows… in comparison [in my home country] they are natural because one prepares them. (Parent in comparison group)

Similar to parents in the intervention group, the act of preparing the beverages at home was preferred regardless of the sugar content of those beverages.

I have avoided buying sodas. Sometimes I make juices, but natural, like the Kool-Aid®. (Parent in comparison group)

### Understanding any changes in psychosocial constructs

Finally, to understand the reported changes in the beverage consumption in both groups, the following topics related to psychosocial constructs were deductively identified following the intervention’s theoretical framework.

#### Knowledge about the benefits of drinking water

Parents in both groups were able to explain the benefits of drinking water. However, parents in the intervention group expanded upon this knowledge by mentioning body’s hydration, energy levels, oral health (for tap water), weight loss and cleansing and healing body organs.

Water maintains [my son] more hydrated, healthier. He has lost around 12 lbs. (Water Up!@Home participant)

Although participants in the comparison group also described that water was cleansing, no other benefits of drinking water were discussed.

Drinking pure water is better because it cleans the organism. (Comparison parent)

#### Knowledge about the health effects of sugar-sweetened beverages

Participants in both groups were able to discuss health effects of SSB consumption on diabetes and obesity, cavities and hyperactivity.

…soda gives you cavities; they damage your teeth…another thing, they can give diabetes to children. (Water Up!@Home participant)

…when they eat too much sugar or juices [the children] are restless (Participant in comparison group)

Since the comparison group did not receive any information from the curriculum, they were asked how they had learned this information. Participants in the comparison group described that they learned about the health risks related to SSB consumption from health and educational professionals or from internet resources.

… sometimes I am in the meetings through the school and there they give us nutrition classes. I have learned from many people, from example the teacher of my son… and through my oldest son’s school there is a nutritionist who gives us classes. (Participant in comparison group)

#### Perceptions of susceptibility to, and severity of, the health consequences of consuming sugar-sweetened beverages

Participants in both groups discussed their personal or familial susceptibilities to, and severity of, the health consequences of SSB, including their and their relatives’ propensity to develop diabetes and other health problems.

In this country there are lots of people, us Hispanics with sugar in the blood, diabetes, I take my child to the clinic and they told me that he was beginning to develop sugar in the blood, and he lost around 30 lbs. (Water Up!@Home participant)

Today diabetes is something I am afraid of… I have seen it in some of my family members. (Participant in comparison group)

#### Skills and self-efficacy to replace sugar-sweetened beverages with water

A major theme that emerged among parents that received the full intervention was that they gained skills to read and interpret nutrition labels at the store to assess the contents of beverages (including sugars) and that this informed their purchasing decisions.

Through the process of seeing, checking how much sugar each thing has, we are no longer consuming as much sugar, we are reducing it; we are replacing it with natural water. (Water Up!@Home participant)

## Discussion

This study sought to understand how the Water Up!@Home intervention (curriculum plus low-cost water filter pitcher) or providing a water filter pitcher only may have led participants in both experimental groups to increase their water and reduce their SSB intake. The explanatory qualitative data suggest that the low-cost water filter may have been enough to stimulate water consumption in both experimental groups by (1) increasing parents’ perception of water safety; (2) acting as a cue to action to drink water; (3) improving the flavour of water (which was linked to perceptions of safety) and (4) increasing the perception that this option was more economical than purchasing bottled water. Safe and palatable drinking water was therefore more accessible and freely available at home, leading participants to not ration their water consumption as before when they relied entirely on purchased bottled water.

In terms of theoretical constructs of the intervention, although parents in the intervention group were able to provide more detail about health consequences of drinking SSB and water, the findings suggest that parents in comparison group already had some knowledge about the health benefits of drinking water and the health consequences of consuming SSB, and they were also concerned about their susceptibility to these health consequences. For example, participants from both groups expressed fear of them or a family member developing diabetes, even though only intervention participants received education about the health consequences of SSB. However, only parents in the intervention group were able to explicitly describe strategies that they learned from the intervention to reduce SSB and juice consumption and skills they gained during the intervention to inform their beverage decisions. In contrast, no major theme emerged among comparison group parents in terms of whether they noticed any changes in their beverage behaviours since participating in the study. In this group, the provision of the water filter alone may have enabled or cued them into action to apply their existing knowledge to drink more water. Perhaps that increased water consumption displaced SSB consumption.

A unique finding from the current study is that participants identified a sense of safety when they could ‘see’ the origin of the source of the tap water (whether in their home countries or the USA). Seeing the water filtration process via the water filter pitcher seemed to have contributed somewhat to this sense of safety, but the negative cases suggest persistent mistrust in tap water in the USA that was not completely addressed by the filter. These descriptions agree with our previous formative work in this community, where tap water in the USA was described as being ‘recycled from the toilet’, in contrast to drinking water in their home countries in Central America because they knew the water source^(24)^. Hispanic households in the USA are among the groups with the lowest use of water filtration systems; this makes sense in view of the theory that people who are seriously concerned about the safety and contamination of tap water consume more bottled water, whereas those who have water filtration systems are only primarily concerned with the taste and organoleptic qualities of drinking water^(37,38)^. This could partially explain why at baseline, participants in the current study only drank bottled water, and why there was persistent mistrust in drinking tap water even after participants had received a water filter. Mistrust in tap water has been attributed to a myriad of interrelated factors, including early life experiences with water insecurity, built and contextual environment and geography, among others^(38,39)^. Mistrust in tap water and the water systems can also reflect larger and historical dynamics of social inequities and mistrust of government institutions^(40)^. As an intervention, our findings from the Water Up!@Home RCT^(31)^ and from the explanatory qualitative interviews reported here suggest that the use of a water filter could help to alleviate water insecurity concerns in some of the participants, although we cannot obviate the possibility that participants accepted the water filter because it came from a trusted source with whom they already had a relationship (i.e. the home visitors from the EHS).

Taken together, these findings contribute to expand the discussion about water safety and security beyond the roles of sanitation and hydration^(41)^: water security and inequities in water systems may be contributing to higher burden of SSB consumption in already vulnerable communities. Although some have posited that consumption of water in place of SSB may not be effective because the two beverages tend to be consumed in different places and at different times of day^(13)^, past interventions that aimed at increasing water access seemed to have an effect on reducing SSB intake^(42)^. The actual mechanisms of replacement or displacement warrant further understanding. In our study, while intervention parents explained that they consciously tried to consume less SSB and more water via various strategies (taught in the intervention), comparison parents simply explained that they drank more water because of the filter. Since the latter also reported significantly lower SSB consumption after the provision of the water filter^(31)^, this seems to suggest that greater access to water that is perceived as ‘drinkable’ can increase water intake and displaced intake of other beverages, such as SSB, perhaps due to body water homoeostasis^(39)^.

Findings from this study also suggest implications for the role of water safety and security on SSB consumption. Displacement of SSB by water could be feasible in this community if water safety and security are addressed. Past interventions that aimed at increasing water access seemed to have an effect on reducing SSB intake^(42)^; however, others have posited that consumption of water in place of SSB may not be effective because the two beverages tend to be consumed in different places and at different times of day^(13)^. In our current study, intervention parents explained that they diluted SSB and juice with water or that they drank water instead of SSB; and comparison parents only stated that they drank more water because of the filter. Whether SSB consumption was displaced as a result of the increased water consumption among comparison families warrants further study.

One final discussion point is related to the concepts of what Latino parents relate to ‘healthy beverages’. Our findings regarding the perception that homemade beverages were natural and healthier than other prepackaged beverages, regardless of actual nutrient content, is congruent with findings published elsewhere among various Latino populations in the USA and in Latin America: a study conducted to understand what influences parents’ perceptions of a ‘healthy’ beverage reported that being homemade, made with fruit and containing vitamins were more influential to the definition of ‘healthy’ among Hispanic parents compared with African-American parents in the USA^(26)^. Other studies have reported that Hispanics identify homemade beverages as healthy alternatives to store-bought beverages, despite sugar content^(43,44)^. Sweet fruit and grain-based beverages (i.e. rice-based drinks *horchatas* or oatmeal-based drinks *avenas*) are ubiquitous in Hispanic communities in the USA and in Latin America; they are often consumed at home but are also popular in independently owned Latino restaurants in the USA^(45)^ and are a major source of energies from added sugars^(46,47)^. These perceptions warrant further understanding as they may hold important information for public health nutrition messaging in these communities.

### Limitations and strengths

Several limitations should be taken into consideration in the interpretation of results. Social desirability bias cannot be ruled out: participants may have answered questions in line with their perceptions of what the interviewer wanted to hear. This type of bias was minimised with careful probing following open-ended questions, by emphasising to the participants that the aim was to understand their previous quantitative responses and that there were no right or wrong answers. Strengths include (1) reaching data saturation in both groups; (2) member checking on findings and (3) using a CBPAR process in the design of this study including the interpretations of these qualitative findings via iterative sessions with the intervention implementers and community partners.

## Conclusions

The results suggest that the water filter facilitated water consumption for all participants; this consumption may have displaced SSB consumption via dilution or full replacement, either as a conscious decision by participants (in the case of the intervention) or an unconscious decision by participants in the comparison group. The findings emphasise the need to understand the role of water safety and security on SSB consumption.
